# 11β Hydroxysteroid dehydrogenase – 1 activity in type 2 diabetes mellitus: a comparative study

**DOI:** 10.1186/s12902-019-0344-9

**Published:** 2019-01-24

**Authors:** Ravindra Shukla, Asish Kumar Basu, Biplab Mandal, Pradip Mukhopadhyay, Animesh Maity, Satyam Chakraborty, Praveen Kumar Devrabhai

**Affiliations:** 1 Department of Endocrinology & Metabolism, AIIMS, Jodhpur, 342005 India; 2 Medical College Kolkata, Kolkata, India; 3 IPGMER, Kolkata, India

**Keywords:** 11 β HSD 1, Type 2 diabetes mellitus, Cortisone acetate test

## Abstract

**Background:**

A comparative study of 11 β HSD 1 activity in type 2 diabetes mellitus subjects with respect to fasting blood glucose and other metabolic parameters was conducted.

**Methods:**

A case control experimental study was performed enrolling thirty type 2 diabetes mellitus patients and thirty age, gender and BMI matched controls using cortisone acetate test.

**Results:**

The rise of serum cortisol after oral 25 mg cortisone acetate from baseline (dexamethasone suppressed level) is higher in subjects with type 2 diabetes and is associated with exercise, BMI, SGOT but not daily calorie intake, lipid parameters and thyroid status. Fasting blood glucose after overnight 1 mg oral dexamethasone is a strong predictor of 11HSD1 activity, irrespective of presence of type 2 diabetes.

**Conclusion:**

11β HSD 1 activity is higher in type 2 diabetes mellitus subjects, especially those who are lean. Future 11 β HSD 1 inhibitors targeting metabolic syndrome, will be most useful in those with increased fasting blood glucose. The role of DHEAS and vitamin D status needs to be explored.

**Electronic supplementary material:**

The online version of this article (10.1186/s12902-019-0344-9) contains supplementary material, which is available to authorized users.

## Background

The enzyme 11 β HSD is a bi-functional enzyme with activity dependent on NAD(H)/NAD ratio. Type 1 activity of 11βHSD implies cortisone (E) to cortisol (F) conversion. Cortisol generated by 11βHSD1 in hepatocytes can increase hepatic glucose output by activating transcription of phosphoenol pyruvate kinase (PEPCK). This increase in hepatic glucose output occurs independent of any interaction with insulin, glucagon or free fatty acids (FFA) [1].Transgenic mice over expressing 11βHSD1 develop metabolic syndrome but not obesity. This has lead to the hypothesis that type 2 diabetes mellitus is a form of localized Cushings syndrome [2].This experimental study compares 11β HSD1 activity between type 2 diabetes mellitus patients and healthy controls. This study also correlates 11βHSD1 activity with various metabolic parameters in each group. We devised a unique protocol of cortisone acetate test to estimate 11βHSD1 activity. The test has been described previously [3].

## Methods

The study was carried out in 2014–2015 and Test protocol as described below was approved by Institutional Ethics Committee (IEC) of Kolkata Medical College, India. The objective was to assess 11βHSD1 activity in cases and compare it with healthy controls. Thirty subjects with type 2 diabetes and thirty healthy controls were selected for the study. Cases were selected from those attending outpatient services of Department of Endocrinology Medical College Kolkata, while healthy controls were selected from those volunteering for the study. The sample size calculated using formulae:

Sample size = Z × S.D./d2 where.

Z = standard normal variate at 1.96.

S.D. = 4.5 μg/dl based on pharmacokinetic absorption data of cortisone acetate.

d = precision = 3 based on previous study on type 1 DM [3] Those with known diabetic complications and HbA1c > 7 mmol/l or random blood glucose > 200 mg/dl in preceding 7 days were excluded. Healthy subjects were chosen from those volunteering for the study.

### Cortisone acetate test protocol

All subjects were admitted a day before testing and were given 1 mg dexamethasone at 11 pm. Next day, 8:00 am fasting blood glucose and serum cortisol sample was taken. Thereafter, tablet cortisone acetate (25 mg) was given with standardized breakfast of two brown bread slices. Second sample of serum cortisol was taken at 8 45 am. The difference between the cortisol values was denoted as delta cortisol. Delta cortisol is two taken as surrogate of 11βHSD1 activity .

Roche COBAS e411 (Co-efficient of variation < 10%) was used for measurements of serum cortisol, TSH, free T4, TT4, 25(OH) vitamin D. CENTAUR was used for lipid profile and other investigations. NHAMS and NIN-ICMR guidelines were followed for anthropometry and daily dietary calorie calculation, respectively. Physical activity, BMI, and calorie intake was categorized (see Additional file 1). Kolmogorov-Smironoff and Shapiro Wilk tests were used to check normality. Students t test, one way & two way ANOVA, Mann-Whitney U test, Friedmen test, Kruskal Wallis tests were used for in between groups comparison. Wilcoxen Signed Rank test was used to compare pre & post dexamethasone fasting blood glucose. The Pearson & Spearmen test was used to test association between variables. A multiple regression analysis model was used to find out various parameters affecting delta cortisol activity. SPSS software version 20 was used for statistical analysis.

## Results

Study subjects were well matched for age & weight (Table 1). Metabolic parameters were compared using t-test and Mann-Whitney test as described in Table 2 . All subjects (except one diabetic subject) had dexamethasone suppressed cortisol level < 1.8 mcg/dl. There was no statistically significant difference between diabetic and non diabetic subjects in 8 00 h serum cortisol level. (*p* = 0.211, Mann-Whitney test) (Table 3). The 11 β HSD1 activity in diabetes subjects was found to be significantly increased (*p* = 0.022, Mann-Whitney test) as compared to controls (Table 3).

**Table 1:** Characteristics of Diabetes and Healthy controls

	cases and control	
	Diabetes	Healthy controls
	Mean	Mean
Age (yrs)	46	40
Daily average calorie intake(kcal)	1876	2172
Height	1.56	1.57
Weight	62	61
Body mass index (kg/m2)	25.25	24.65
Waist circumference (cm)	89	83
Systolic BP (mm of Hg)	137	125
Diastolic BP(mm of Hg)	78	75

Subjects were well matched in mean age, BMI and waist circumference. Age range was (32 years −62 years) and (20 years − 64 years) in cases and control respectively. Diabetics had lower total calorie intake and higher systolic blood pressure. Mann-Whitney test was used for comparison

**Table 2:** Fasting lipid profile, liver & renal function tests of the study subjects

Lipid profile by automated analyser or ELISA method. All other tests by ELISA	cases and control				*p*-value
	diabetes		healthy controls		
	Mean	Standard Deviation	Mean	Standard Deviation	
total cholesterol (mg/dl)	171	35	185	42	0.84
LDL (mg/dl)	107	30	120	27	0.08
HDL (mg/dl)	41	10	41	10	NS
Total triglyceride (mg/dl)	151	50	143	49	0.65
Total bilirubin (mg/dl)	1.0	.4	.9	.1	0.4
SGOT (U/L)	41	16	34	20	0.029
SGPT (U/L)	36	14	31	11	0.20
S. Albumin (U/L)	4.5	.7	4.7	.4	0.069
S.Globulin (U/L)	3.3	.6	3.0	.0	0.07
Alkaline phosphatase	163	62	103	67	,0.01
S.creatinine (mg/dl)	.9	.1	.9	.1	NS
25 (OH) vitamin D	18.4	7.4	23	12.3	0.021

t-test was done for parametric variables (Total Cholesterol, LDL,HDL,Triglycerides, alkaline phosphatase)

Mann-Whitney test was done for non-parametric variables (Bilirubin, SGOT<SGPT,Albumin, Globulin,creatinine, 25(OH)vitamin D)

Diabetic subjects have higher mean Alkaline phosphatase (ALP),Serum Glutamate oxaloacetate transferase (SGOT), Serum Glutamate Pyruvate Transferase (SGPT) and lower 25(OH) vitamin D, but better mean lipid parameters as compared to controls

**Table 3:** Hormonal evaluation of study subjects

Delta cortisol is difference in S.cortisol levels before and 40 min after Tablet cortisone acetate 25 mg PO	cases and control				*p*-value
	Diabetes		Healthy controls		
	Mean	Standard Deviation	Mean	Standard Deviation	
TSH (mU/L)	2.65	1.18	2.36	1.13	0.11
free T4 (ng/dl)	1.2	.3	1.1	.2	
Fasting blood glucose (mg/dl)	150.43	40.39	83.37	7.80	< 0.01
8 am cortisol (mcg/dl)	1.29	2.19	.84	.46	0.211
8 40 am cortisol (mcg/dl)	13.58	10.38	7.52	7.24	0.012
delta (8 40–8 00 am cortisol)	12.29	10.65	6.68	7.24	0.022

Mann-Whitney test used to compare values in diabetics and healthy control

Diabetics had higher mean Fasting blood glucose(FBG),TSH & cortisol values

**Fig. 1:**
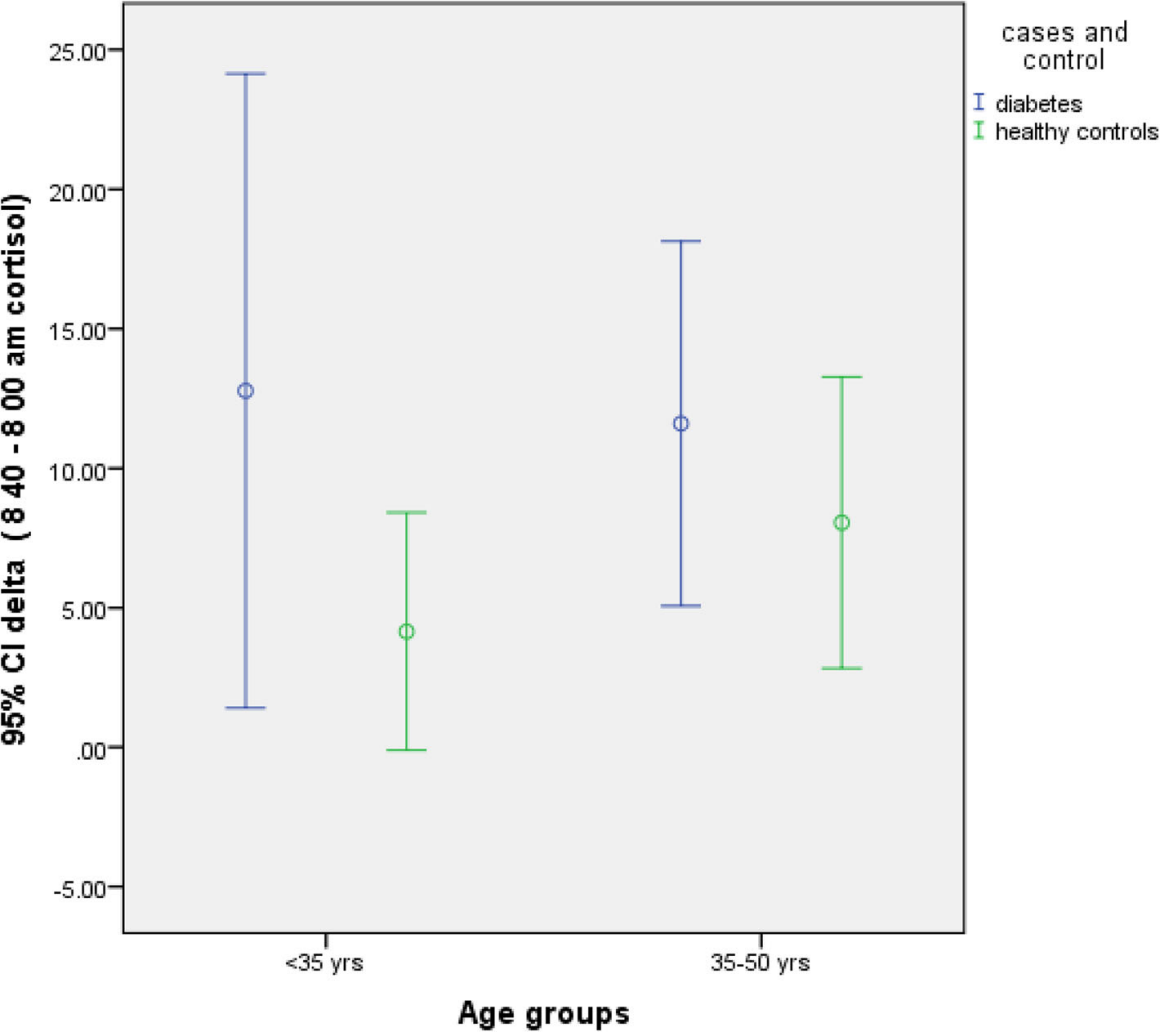
Delta cortisol according to age groups. 11β HSD1 activity was increased in young(< 35 years) diabetics but not older (> 35 years), when compared to healthy controls

**Fig. 2:**
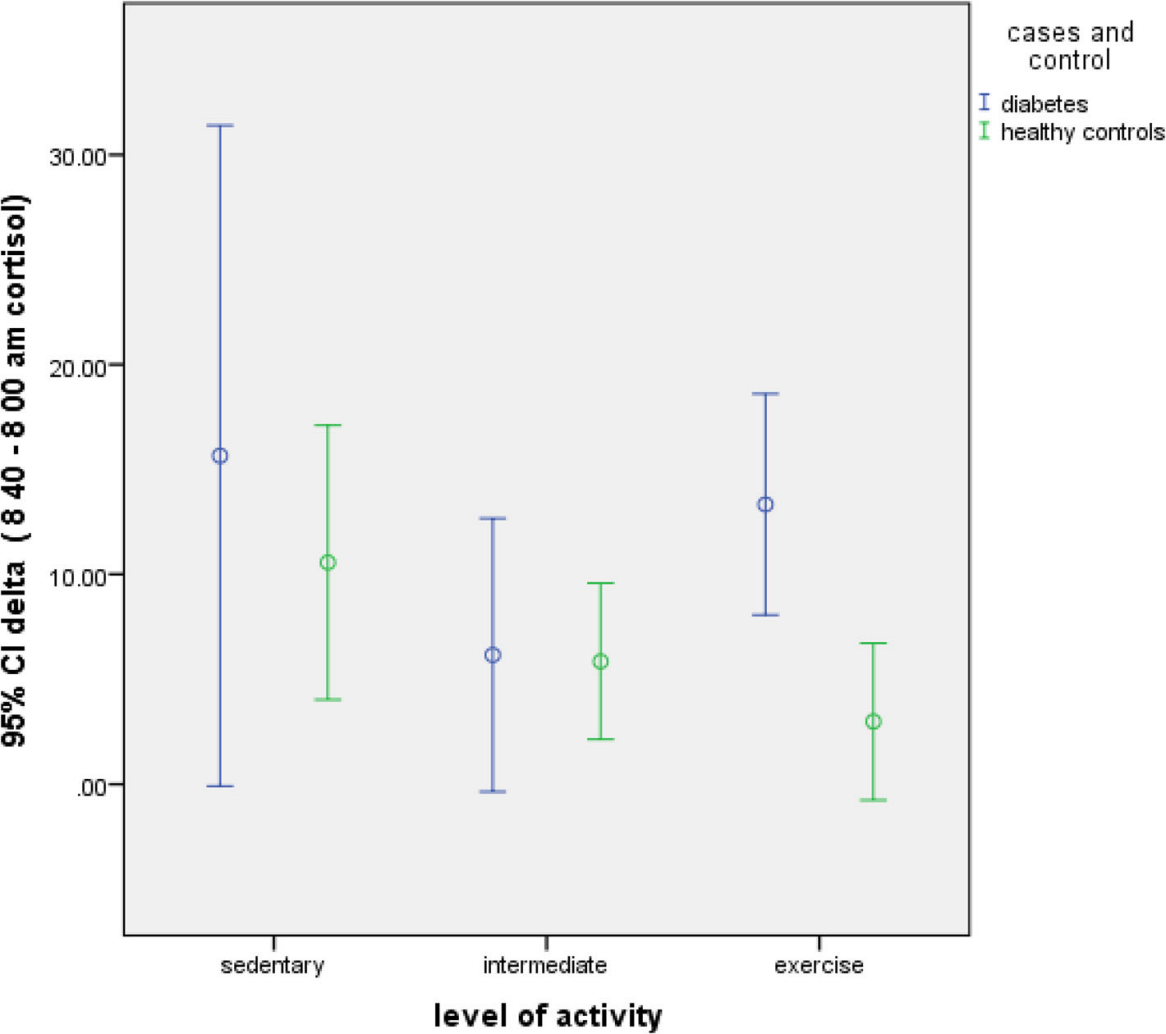
Delta cortisol in those who exercise. Diabetics who exercise > 180 min/week have higher 11β HSD1 activity

**Fig. 3:**
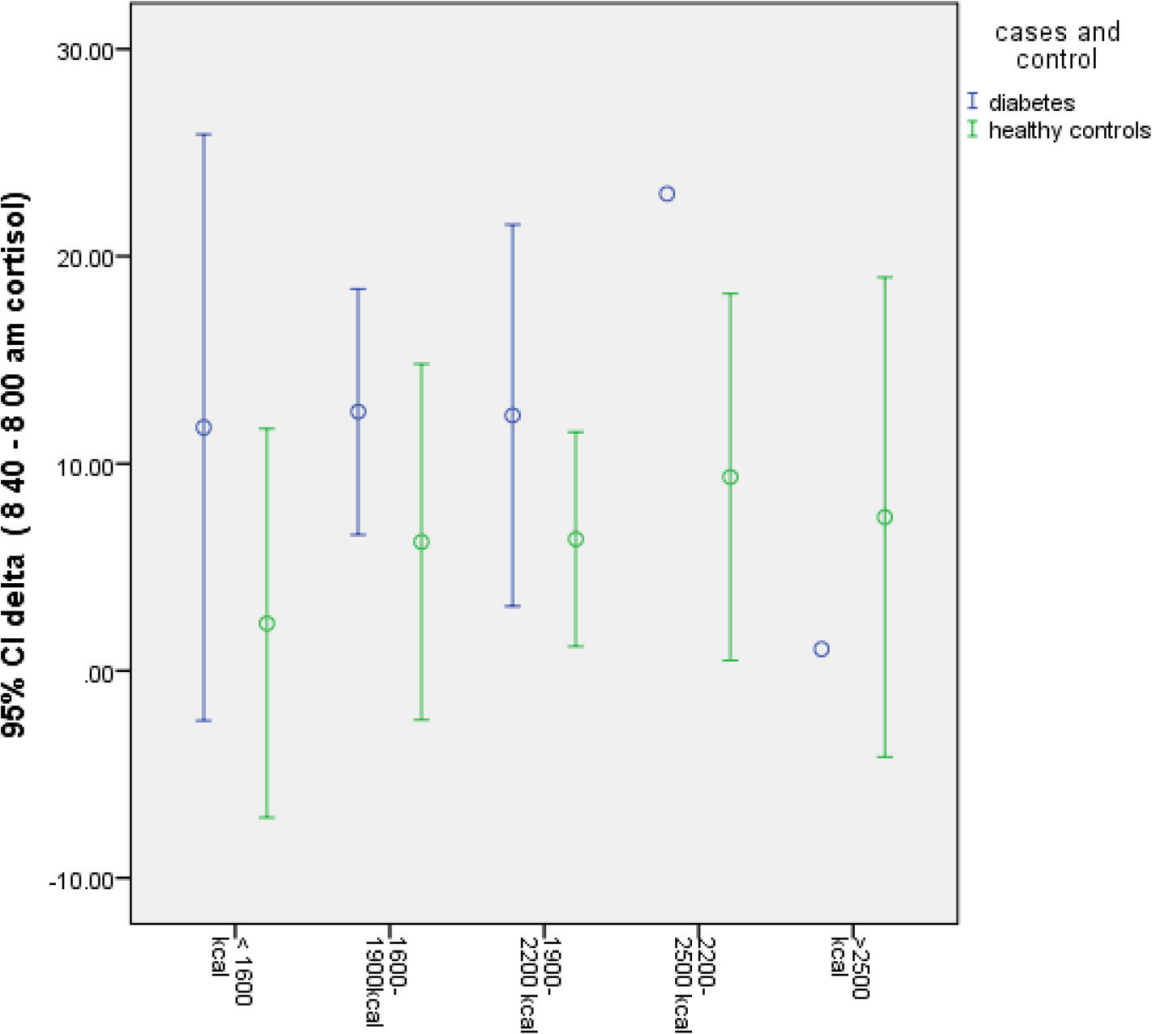
Delta cortisol according to calorie groups. Higher 11β HSD1 activity seen in diabetics with lower calorie intake, but was not statistically significant when Kruskal Wallis test was applied (*p* = 0.08)

In study subjects < 35 years of age, 11β HSD1 activity was significantly increased in diabetics as compared to healthy controls (Table 4, Fig. 1) [*p* = 0.005, Mann-Whitney test]. In other age groups, 11β HSD1 activity was not statistically different between diabetics and controls (Table 4, Fig. 1). Similarly, in subjects who exercise > 180 min/week, 11β HSD1 activity was found to be significantly more in diabetics (Table 4, Fig. 2) (*p* = 0.004, Mann-Whitney test). Diabetes subjects with BMI < 23 kg/m2 had significantly higher 11β HSD1 activity as compared to healthy controls (Table 4) (*p* = 0.017, Mann-Whitney test). There was no effect of calorie intake on 11β HSD1 activity (Table 4, Fig. 3) .

**Table 4:** Comparison of delta cortisol in diabetes subjects and control

factor	Delta cortisol Cases (mcg/dl)	Delta cortisol control (mcg/dl)	*P* value
Age (n)			
< 35 (20)	13	5.4	0.005
35–50 (31)	12	9	0.08
> 50 (08)			-
BMI (n)			
< 23 (19)	14.2	8.9	0.017
23–27 (29)	11.3	9.1	0.142
> 27 (12)	9	8.2	0.755
Physical activity(n)			
Sedendary (15)	16	11	0.06
Intermediate(18)	13.4	10.2	0.083
Exercise (27)	15.1	6.2	0.004
Calorie intake			
< 1600 (8)	11.2	6.1	0.052
1600–1900 (18)	12.3	8.2	0.08
1900–2200 (21)	12	8.2	0.03
2200–2500 (7)	9	8.4	0.3
> 2500 (6)	8	8.3	0.24

Mann-Whitney test was used to compare delta cortisol between cases and control as shown above

Delta cortisol was increased in diabetics as compared to healthy controls and was statistically significant in those subjects < 35 years of age (*p* = 0.005), BMI < 23 (*p* = 0.017) and in those who exercise (*p* = 0.004)

When delta cortisol was compared in various calorie groups, it was significantly more in diabetics. However, when Kruskal Wallis test applied, the difference was not significant. Also see Fig. 3

11β HSD1 activity was inversely associated with waist circumference (*p* = 0.027, rho = − 0.356, Pearsons correlation) in diabetics, albeit weakly. SGOT was significantly increased in diabetes and was strongly associated with 11β HSD1 activity in diabetes subjects {spearman rho = 0.638, *p* = 0.002). Fourteen diabetes subjects had finding of fatty liver on sonography. Post dexamethasone FBG was significantly raised in subjects with diabetes (*p* = 0.01, Wilcoxen Signed Rank test) but not healthy controls. 11β HSD1 activity was strongly correlated with post dexamethasone FBG in both cases and controls. Even after adjusting for increased FBG in diabetics, post dexamethasone FBG correlated with delta cortisol. Curiously, the same association was not observed with pre dexamethasone FBG. LDL was significantly associated with 11β HSD1 activity in healthy controls but not diabetics.

11βHSD1 activity was analysed separately in diabetes and healthy participants using Spearman correlation (Table 5). In diabetics, 11βHSD1 activity was found to be associated with SGOT(*p* = 0.02), alkaline phosphatase (ALP) (*p* = 0.03), FBG(*p* = 0.008). In healthy subjects, it was found to be associated with systolic blood pressure (*p* = 0.013), LDL (*p* = 0.006), triglycerides. Age, BMI, TSH and daily calorie intake had no effect on 11βHSD1 activity. Multiple regression analysis was performed to predict 11β HSD1 activity using automatic linear modeling, with 11β HSD1 activity being taken outcome variable using forward stepwise model building method. Five variables were identified at *p* < 0.05 (Table 6).

**Table 5:** Delta cortisol analysed separately for cases and control

	Case *p*-value	Controls *p*-value
SBP	0.235	0.013
DBP	0.101	0.007
LDL	0.113	0.006
Triglycerides	0.8	0.6
FBG	0.008	0.02
Alkaline Phosphatase	0.03	0.4

SBP, DBP, LDL, Triglycerides, FBG,Alkalile phosphatase was analysed for correlation with 11 β HSD 1 activity in diabetics (1st column). Spearman correlation was used

SBP, DBP, LDL, Triglycerides, FBG,Alkalile phosphatase was analysed for correlation with 11 β HSD 1 activity in healthy controls (2nd column). Spearman correlation was used

**Table 6:** Results of linear regression using automated linear model

Variable	coefficient	importance	significance
FBG	0.164	0.502	0.000
SGOT	0.194	0.149	0.007
Pre FBG	0.206	0.018	0.018
SGPT	0.231	0.026	0.026
1 ÷ vitamin D	0.230	0.023	0.033

The model assumes delta cortisol to be gold standard of 11 β HSD1 activity .5 variables were identified at *p* < 0.05. -FBG, SGOT, pre dexa FBG, SGPT and inverse of total 25 (OH) vitamin D. At *p* < 0.1 systolic blood pressure was also identified as predicting variable (not shown)

## Discussion

Cortisone acetate test has been previously described for measuring 11β HSD1 activity [3].We added overnight dexamethasone suppression, so as to bring 0800 h basal cortisol at similar levels in the study subjects. It has been shown that serum cortisol levels attained 45 min after cortisone acetate ingestion is entirely due to 11 β HSD1 activity [4]. Hence, endogenous cortisol secretion acting as confounder, is ruled out. Delta cortisol can thus be used as surrogate marker of 11βHSD1 activity in splanchanic circulation, more specifically liver [5].

Increased 11βHSD activity could lead to development of metabolic syndrome [6].11 β HSD knock off transgenic mice have higher insulin sensitivity. They are also resistant to develop metabolic syndrome when put on high fat diet [7]. An 11 β HSD1 inhibitor has been shown to reverse metabolic syndrome in mice over expressing 11βHSD1 [8].This has lead to the hypothesis that increased 11 β HSD1 activity has a contributory role in development of type 2 diabetes/metabolic syndrome. A study carrying out cortisone acetate test in young type 1 diabetes subjects reported decreased 11βHSD 1 activity and inverse correlation was found with insulin dose requirement. The decreased 11 β HSD1 was attributed to insulin [4] .Insulin directly inhibits 11 β HSD1 activity in liver [9].Insulin resistance would similarly increase 11 β HSD 1 activity. It would be imperative to compare our results with those studies that evaluated 11 β HSD by other methods e.g. Urinary tetrahydrocortisol (THF) + allo-THF: tetrahydrocortisone (THE) ratio [10]. An increased ratio means relative decrease in cortisol inactivation i.e. increased 11β HSD1. Studies using (THF + allo-THF): THE ratio have not found any difference with respect to type 2 DM [11] except one [12]. However, the method of using THF_allo-THF: THE ratio has an inherent fallacy. The cortisol- cortisone metabolism is likely affected by differences in 5alfa/5 β reductase enzyme activities and this can lead to erroneous conclusion [12]. One study using cortisone acetate test to estimate 11βHSD1 activity in type 2 diabetes found it to be decreased, but baseline cortisol was not suppressed [6]. Studies examining 11βHSD activity in diabetes patients by 11 β HSD1 mRNA in skeletal tissue have found it to be increased in diabetes [13]. Previous report of decreased post dexamethasone 9 00 h cortisol as compared to controls [6] has not been confirmed in our study, possibly because we used 1 mg instead of 0.25 mg dexamethasone.

In humans, the 11 β HSD1 activity differs with age. Cortisone acetate is ineffective in treating congenital adrenal hyperplasia (CAH) in children less than 2 years of age, suggesting decreased 11βHSD1 activity in younger age [14]. Growth hormone (GH) inhibits 11βHSD1 and with decreasing GH levels, 11β HSD1 activity is increased in elderly [15]. 11βHSD1 activity was compared in three age groups: < 35 years, 35–50 yrs., > 50 yrs. These groups were determined in taking into consideration DHEAS levels, which vary greatly in age groups defined above. DHEAS is an inhibitor of 11βHSD1 enzyme in human adipocytes in vitro [16] . In age group < 35 years, diabetics had higher 11βHSD1 activity as compared to healthy individuals. This was not due to duration of diabetes, as there was no significant difference in 11β HSD 1 activity with diabetes duration. DHEAS levels are higher in young adults and decline precipitously in third decade (so called adrenopause). We speculate that decreased DHEAS levels/ premature adrenopause has lead to increased 11β HSD1 activity in diabetics < 35 years of age. As there is no precedent study examining 11β HSD1 with DHEAS, the matter cannot be concluded. 11 βHSD1 in vivo shows sexual dimorphism with males having higher higher activity [17]. We did not find any difference, possibly due skewed gender distribution of study subjects.

### Effect of exercise

11 β HSD1 activity was significantly increased in those diabetes subjects, who exercise as compared to healthy subjects with same range of physical activity. However, in those with sedentary or lower level of physical activity, no difference in 11βHSD1 activity was observed. Acute intense physical activity leads to increased 11βHSD1 activity [18].The effect of chronic physical activity on 11βHSD1 has been less adequately studied. Chronic physical exercise induces Nuclear Factor kappa B (NFkB) activation and increased transcription in adult males [19]. NFkB has been found to induce 11βHSD1 activity under hypoxic conditions [20].Increased cellular hypoxia can thus lead to increased 11βHSD1 activity. Exercise induces hypoxia in skeletal muscle due to increased demand for ATP. It can be therefore hypothesized that diabetic subjects had ‘maladapted’ cellular energetics leading to increased 11 β HSD1 activity on > 180 min/week of exercise. Increased mitogenesisis of arterial smooth muscle cell is a feature of insulin resistense. It is accompanied by increased NFkB activity and higher SBP [21] . Diabetic subjects who exercised had increased systolic blood pressure (SBP) as compared to controls. This paradox of increased blood pressure with exercise can be explained by NFkB induced 11β HSD1 activity .

Both BMI and waist circumference were well matched in cases and controls. Diabetes subjects with normal weight (i.e < 23 kg/m2) were found to have significantly higher 11β HSD1 activity not only compared to controls, but also compared with those diabetics with BMI > 23 kg/m2. As no standards for ‘normal’ delta cortisol exist, the above statement could mean decreased 11 β HSD1 activity in healthy subjects rather than increased activity in diabetics. Indeed decreased 11 β HSD1 activity has been postulated as an adaptation to protect from adverse metabolic effect of obesity in Zucker diabetic rats [22]and possibly in humans [11]. Those who fail to downregulate 11βHSD1 activity develop diabetes, hypertension and dyslipidemia. Decreased 11βHSD1 activity might protect healthy controls from diabetes. However, this adaptation was lost at BMI > 23. At BMI greater than 23, 11βHSD1 activity was similar in cases and control. This indicates that after a certain degree of obesity (which in our population is BMI > 23), other factors i.e. insulin resistance take precedence in causation of diabetes, hypertension and dyslipidemia. Atleast one selective 11 β HSD1 inhibitor has entered phase 3 trial [23]. On basis of this study,a hypothetical target group for 11 β HSD1 inhibitor would be those with BMI < 23 kg/m2 - The “thin fat Indian” phenotype.

Waist circumference was inversely correlated with 11βHSD1 activity in diabetes i.e. increased waist circumference was associated with decreased 11 β HSD1 activity. Waist circumference represents visceral adipose tissue (VAT) more closely. The VAT has almost exclusively cortisol (F) to cortisone(E) conversion (i.e. 11β HSD2 actvity). As a result the substrate cortisone (E) is generated through VAT is presented to liver for localized cortisol generation [5]. 11 β HSD, too, is located around portal venules in the liver [24]. However in subjects with higher VAT, there is relative increase in 11β HSD1 activity of visceral fat and decrease in liver 11 β HSD1 activity. This “compensatory” decrease in liver 11βHSD1 activity is what our test measured. It could be due to adipokines [6]. In those with gynoid fat distribution visceral tissue 11βHSD1 is decreased [25].

Both systolic and diastolic blood pressure was associated with increased 11 β HSD1 activity in controls but not in diabetics, possibly because of confounding effect of anti-hypertensives. Cortisol- cortisone shuttle regulated by 11 β HSD1 activity plays role in increased vascular tone, more so in clinical states of primary aldosteronism (where diastolic hypertension is prominent) and Cushing syndrome [26]. Mice over expressing 11βHSD1 have severe hypertension due to increased angiotensinogen generation by the liver [27] . Our study finds the association for the first time in normotensive healthy adults.

Two FBG values were available for each patients. Matching FBG could not be possible in two groups. Dexamethasone significantly increased FBG in diabetic patients but not healthy controls. 11 β HSD1 activity was strongly associated with FBG in both cases and control. Linear modeling showed that even after adjusting for the presence of diabetes, post dexamethasone FBG was associated with increased 11 β HSD1 activity. The same association was not found with pre dexamethasone FBG. This points to a possible influence of dexamethasone itself on 11βHSD1 activity. Dexamethasone has been shown to induce 11β HSD 1 gene transcription both directly and indirectly. Direct action takes days, while indirect action takes minutes to manifest. Indirect action occurs by binding to P2 promotor region, located upstream to HSD11B1 gene [28, 29].

LDL was well matched in cases and controls. But only in healthy subjects, 11 HSD1 activity was associated with LDL. We attribute lack of association of 11 β HSD1 activity with LDL in diabetes subjects to concomitant statin use. Since statins suppress low grade inflammation of chronic diseases, they could well have inhibitory effect on 11 β HSD1 activity. We could not relate 11β HSD1 activity with triglycerides. Glucocorticoids increase total cholesterol, LDL, HDL, but major increase is in triglycerides [30].Thus, there is diversion between glucocorticoid (GC) receptor action and 11βHSD1 activity on lipid metabolism. This can be explained by non-genomic action of glucocorticoids on TG synthesis pathway, circumventing 11 β HSD enzyme. NAFLD is widely considered as hepatic manifestation of metabolic syndrome. Previous studies have documented derangement of cortisol metabolism, i.e. increase localized liver glucocorticoid action [31]. Systemic hypercortisolemia of Cushings disease ironically protects from hepatic steatosis, and glucocorticoids have therapeutic benefit in alchoholic hepatitis. Altered 11βHSD1 activity might be one of the reason [32]. SGOT was strongest predictor of 11 β HSD1 activity on multiple regression. Since SGOT/SGPT ratio is increased in non alchoholic steatohepatitis (NASH), we speculate role of 11 β HSD1 activity as determining factor in fatty liver to NASH progression.

Although serum alkaline phosphatase (ALP) was significantly increased in diabetics, no association with 11 β HSD1 activity was found after multiple regression. The 11β HSD1 activity is present in both osteoclasts and osteoblasts. Invitro studies have shown increased 11 β HSD1 activity to be associated with bone reabsorption markers including ALP [33]. Firstly, increased ALP levels in diabetics can be due to more prevalent vitamin D deficiency [34, 35].Secondly, although ALP is bone turnover marker and bone turnover markers are associated with 11βHSD1 activity, it is premature to draw any conclusion in the absence of other bone turnover markers. Thirdly, increased ALP in diabetics who undertake rigorous physical activity might be due to subtle trauma, microfractures and osteoarthritis. Fourthly, the test was not for bone specific ALP. TSH was evaluated in the two the groups and no significant association was found with 11βHSD1 activity.

## Conclusion

We compared 11β HSD1 activity in type 2 diabetes subjects with healthy controls using a novel protocol of cortisone acetate test. 11βHSD1 activity is increased in type 2 diabetes mellitus, those with diabetics with BMI < 23 kg/m2, and those who engage in regular exercise. It is also associated with hepatic enzyme elevation,systolic blood pressure and possibly with DHEAS and vitamin D deficiency. There exists a dichotomy between glucocorticoid action and 11β HSD1 activity in lipoprotein metabolism. Fasting blood glucose, especially after overnight dexamethasone is a strong determinant of 11β HSD1 activity and can be used as its surrogate marker. Future 11βHSD1 inhibitors targeting metabolic syndrome will be most useful in those with increased fasting blood glucose. .

## Additional file

Additional file 1: Categorization of study subjects (DOCX 13 kb)

## Abbreviations

ALP: Alkaline phosphatase
BMI: Body Mass Index
DHEAS: Dihydroxyepiandrostenedionesulphate
FBG: Fasting blood glucose
HSD: Hydroxysteroiddehydrogenase
NAD (P): Nicotinamideadenine dinucleotide phosphate
NAD: Nicotinamide adenine dinucleotide
NAFLD: Nonalchoholic fatty liver disease
NFkB: Nuclear Factor kappaB
PEPCK: Phosphoenol pyruvate kinase
SGOT: SerumGlutamate oxaloacetate transferase
SGPT: Serum Glutamate PyruvateTransferase
TG: Triglycerides
THE: Tetrahydrocortisone
THF: Tetrahydrocortisol
TSH: Thyroid Stimulating Hormone
VAT: Visceral Adipose Tissue
WC: Waist circumference
