# Supplementary material for: 11β Hydroxysteroid dehydrogenase – 1 activity in type 2 diabetes mellitus: a comparative study
Source: BMC Endocr Disord. 2019 Jan 24;19:15. doi: 10.1186/s12902-019-0344-9 (PMC6345010; doi:10.1186/s12902-019-0344-9)
Supplement: Supplementary file 1 — Categorization of study subjects (DOCX 13 kb) [file 12902_2019_344_MOESM1_ESM.docx]

File name: Categorization of study subjects

|  | Title | Description |
| --- | --- | --- |
| A | Categorization of BMI (kg/m2) in 3 groups | normal <23  overweight 23-27  obese >27 |
| B | Categorization of physical activity in 3 groups | sedentary : defined as activities of daily living only; no manual work  exercise : > 180 min /week of exercise    intermediate : < 180 min/week of exercise |
| C | Categorization of daily calorie consumption in 5 groups | \| < 1600 kcal \| \| --- \| \| 1600-1900kcal \| \| 1900-2200 kcal \| \| 2200-2500 kcal \| \| >2500 kcal \| |
